# Regulation of protein degradation pathways by amino acids and insulin in skeletal muscle of neonatal pigs

**DOI:** 10.1186/2049-1891-5-8

**Published:** 2014-01-17

**Authors:** Agus Suryawan, Teresa A Davis

**Affiliations:** 1 United States Department of Agriculture/Agricultural Research Service Children’s Nutrition Research Center, Department of Pediatrics, Baylor College of Medicine, Houston, TX 77030, USA

**Keywords:** Amino acids, Autophagy, Insulin, Leucine, Muscle, Neonate, Protein degradation, Protein synthesis, Swine, Ubiquitin

## Abstract

**Background:**

The rapid gain in lean mass in neonates requires greater rates of protein synthesis than degradation. We previously delineated the molecular mechanisms by which insulin and amino acids, especially leucine, modulate skeletal muscle protein synthesis and how this changes with development. In the current study, we identified mechanisms involved in protein degradation regulation. In experiment 1, 6- and 26-d-old pigs were studied during 1) euinsulinemic-euglycemic-euaminoacidemic, 2) euinsulinemic-euglycemic-hyperaminoacidemic, and 3) hyperinsulinemic-euglycemic-euaminoacidemic clamps for 2 h. In experiment 2, 5-d-old pigs were studied during 1) euinsulinemic-euglycemic-euaminoacidemic-euleucinemic, 2) euinsulinemic-euglycemic-hypoaminoacidemic-hyperleucinemic, and 3) euinsulinemic-euglycemic-euaminoacidemic-hyperleucinemic clamps for 24 h. We determined in muscle indices of ubiquitin-proteasome, i.e., atrogin-1 (MAFbx) and muscle RING-finger protein-1 (MuRF1) and autophagy-lysosome systems, i.e., unc51-like kinase 1 (UKL1), microtubule-associated protein light chain 3 (LC3), and lysosomal-associated membrane protein 2 (Lamp-2). For comparison, we measured ribosomal protein S6 (rpS6) and eukaryotic initiation factor 4E (eIF4E) activation, components of translation initiation.

**Results:**

Abundance of atrogin-1, but not MuRF1, was greater in 26- than 6-d-old pigs and was not affected by insulin, amino acids, or leucine. Abundance of ULK1 and LC3 was higher in younger pigs and not affected by treatment. The LC3-II/LC3-I ratio was reduced and ULK1 phosphorylation increased by insulin, amino acids, and leucine. These responses were more profound in younger pigs. Abundance of Lamp-2 was not affected by treatment or development. Abundance of eIF4E, but not rpS6, was higher in 6- than 26-d-old-pigs but unaffected by treatment. Phosphorylation of eIF4E was not affected by treatment, however, insulin, amino acids, and leucine stimulated rpS6 phosphorylation, and the responses decreased with development.

**Conclusions:**

The rapid growth of neonatal muscle is in part due to the positive balance between the activation of protein synthesis and degradation signaling. Insulin, amino acids, and, particularly, leucine, act as signals to modulate muscle protein synthesis and degradation in neonates.

## Introduction

Skeletal muscle represents 40–50% of body mass in mammals and is a critical regulator of overall metabolism [1]. Therefore, an understanding of the processes involved in the postnatal increase in muscle mass, with associated accumulation of protein, is fundamental [2]. We have demonstrated that protein deposition is high in skeletal muscle of neonatal pigs and decreases with age [3]. This rapid gain in skeletal muscle mass in the neonate is in part due the marked increase in protein synthesis after a meal. The feeding-induced stimulation of muscle protein synthesis is independently regulated by the rise in insulin and amino acids, especially leucine [4,5]. Although muscle protein deposition depends on the balance between the rates of protein synthesis and degradation, less is known of the mechanisms that regulate protein degradation in skeletal muscle of the neonate.

The molecular mechanisms by which insulin and amino acids regulate protein synthesis have been the subject of much investigation [6,7]. Over the past few years, we [8,9] have intensively studied the effect of the post-prandial rise in insulin and amino acids on the activation of signaling components leading to protein synthesis in skeletal muscle of the neonate. We have shown that the stimulation of mammalian target of rapamycin (mTOR) by insulin and amino acids is enhanced in skeletal muscle of the neonatal pig and decreases with development [8]. Activation of mTOR induces the phosphorylation of 4E-binding protein 1 (4EBP-1) and ribosomal protein S6K1 (S6K1), both of which regulate mRNA binding to the 43S pre-initiation complex. However, the developmental changes in the response to amino acids and insulin on the abundance and the activation of ribosome protein S6 (rpS6) and eukaryotic initiation factor 4E (eIF4E) in skeletal muscle has not been determined. Studies using *in vitro* and *in vivo* methods indicate that rpS6 and eIF4E are crucial for the regulation of protein synthesis [10-13]. Of the amino acids, leucine is the most effective in acting as a nutrient signal to activate protein synthesis in skeletal muscle of the neonatal pig [14].

The ubiquitin-proteasome system (UPS) and autophagy-lysosome system are major pathways that are involved in the regulation of protein degradation in skeletal muscle [15]. The autophagy-lysosome system plays a significant role in bulk proteolysis while the UPS is responsible for the control of the degradation of specific proteins [16]. UPS-dependent protein degradation is highly regulated [17]. In this system, lysyl residues of the target proteins are serially attached by ubiquitin (a 76-amino acid protein) which marks them for protein degradation in the proteasome. It is known that two major muscle-specific E3 ubiquitin ligases, MuRF1 (muscle RING-finger protein-1) and atrogin-1 (MAFbx), are important components of the UPS [17].

It has become increasingly evident that autophagy and the UPS are needed for normal muscle development [18]. Although in both systems free amino acids can be generated, only the autophagy system appears to be physiologically regulated by amino acids [16]. Autophagy is a tightly regulated process that involves the degradation of cell components including proteins through the lysosomal machinery [19]. In normal physiological conditions, autophagy is active and plays an important role in several biological processes including cell development [20]. Autophagy is crucial for the survival of neonatal animals under starvation conditions [21] and is induced by early weaning in the piglet model [22]. In the lysosomal degradation pathway, there are two major processes: macroautophagy and chaperone-mediated autophagy (CMA). While the microtubule-associated protein 1 light chain 3 (LC3) is an important component or a marker for macroautophagy, lysosome-associated membrane protein-2 (lamp-2) is crucial for CMA processes [23,24]. mTOR plays a crucial role in the regulation of autophagy via unc51-like kinase 1 (UKL1), an upstream component of LC3 [25]. When the activation of mTOR is high, such as under nutrient sufficiency, mTOR prevents the activation of ULK1 by phosphorylating ULK1 at Ser^757^ resulting in the suppression of autophagy [26].

Studies show that both insulin/IGF-I (insulin-like growth factor-I) and amino acids regulate protein synthesis [6,7,27] and protein breakdown [16,28], however, the role of amino acids on the latter process is not well understood [29]. *In vivo* and *in vitro* studies have shown that the branched-chain amino acids, especially leucine, attenuate muscle protein degradation [30,31]. However, the detailed molecular aspects of the amino acid-induced reduction of proteolysis in skeletal muscle through UPS and autophagy have not been elucidated [16].

The objective of this study was to determine the effects of the postprandial rise in amino acids and insulin on the regulation of specific signaling components involved in protein degradation, and for comparison, protein synthesis, in skeletal muscle of neonatal pigs and how these are modulated by development. We further sought to identify the response of these intracellular signaling components to more prolonged leucine administration. To achieve this, 6- and 26-d-old pigs were infused with amino acids or insulin to attain post-prandial levels for 2 h in Experiment 1. In Experiment 2, 5-d-old pigs were infused for 24 h with physiological levels of leucine, without or with amino acid replacement to prevent leucine-induced hypoaminoacidemia.

## Material and methods

### Animals and housing (experiment 1 and 2)

Multiparous cross-bred (Landrace × Yorkshire × Duroc × Hampshire) pregnant sows (Agriculture Headquarters, Texas Department of Criminal Justice, Huntsville, TX) were housed in lactation crates in environmentally controlled rooms prior to farrowing. Commercial diet (no. 5084; PMI Feeds, Richmond, IN) and water ad libitum were provided. After farrowing, piglets remained with the sow but were not allowed access to the sow’s diet. Three to four d before study, minor surgery was conducted to insert sterile catheters into the jugular vein and carotid artery [32]. The protocol was approved by the Animal Care and Use Committee of Baylor College of Medicine and was conducted in accordance with the National Research Council’s *Guide for the Care and Use of Laboratory Animals*.

### Experimental design

#### Experiment 1

Piglets at 6 d of age (1.9 ± 0.3 kg body weight) and at 26 d of age (5.3 ± 0.8 kg body weight), following an overnight fast, were randomly assigned to one of three treatment groups (*n* = 4–6 per treatment group): *1*) euinsulinemic-euglycemic-euaminoacidemic conditions (C), *2*) euinsulinemic-euglycemic-hyperaminoacidemic clamps (AA), and *3*) hyperinsulinemic-euglycemic-euaminoacidemic clamps (INS), as previously described [33]. During the experiment, blood samples were collected and immediately analyzed for glucose (YSI 2300 STAT Plus; Yellow Springs Instruments, Yellow Springs, OH) and total branched-chain amino acids (BCAA) by rapid enzymatic kinetic assay to establish the basal concentrations of blood glucose and plasma branched-chain amino acids to be used in the clamp technique. Clamps were initiated with a primed, constant (12 mL/h) infusion of insulin (Eli Lilly, Indianapolis, IN) at 0 or 100 ng/kg^0.66^ • min given to attain plasma insulin concentrations of 3 (fasting insulin level) or 30 μU/mL (fed insulin level) and sustained for a period of 2 h. In order to clamp glucose and amino acids at fasting levels, venous blood samples were obtained every 5 min and immediately analyzed for glucose and BCAA concentrations. We adjusted concentrations within ± 10% of the basal fasting concentrations. To obtained euaminoacidemic conditions, the infusion rate of a balanced amino acid mixture was adjusted to maintain plasma BCAA within 10% of fasting levels. Likewise, hyperaminoacidemic conditions were obtained by infusion of a balanced amino acid mixture [34] to raise plasma BCAA concentrations by two-fold the fasting level to reproduce the level of amino acids present in the fed state. To determine circulating insulin concentration, blood samples also were taken at intervals. We achieved the desired substrate and hormone concentrations targeted during our clamp procedure as described in our previous publication [33]. Plasma amino acids levels were raised two-fold to fed levels in the hyperaminoacidemic group and plasma insulin levels were raised to the fed level (~30 μU/mL) in the hyperinsulinemic group. Circulating amino acids, insulin, and glucose concentrations levels were maintained at baseline fasting levels during euaminoacidemia, euinsulinemia, and euglycemia, respectively.

#### Experiment 2

Overnight fasted 5-d-old piglets (2.6 ± 0.1 kg) were randomly assigned to one of three treatment groups (*n* = 6/group) and studied during 1) euinsulinemic-euglycemic-euaminoacidemic-euleucinemic conditions (control, C), euinsulinemic-euglycemic-hypoaminoacidemic-hyperleucinemic clamps (L), and euinsulinemic-euglycemic-euaminoacidemic-hyperleucinemic (L + AA) clamps for 24 h [35]. Animals assigned to the C group were infused with sterile saline at 10 mL/h throughout the infusion period to achieve fasting levels of leucine. Piglets assigned to the L group were infused with leucine at 400 μmol/kg•h to raise circulating levels to that of pigs fed a high protein diet. Pigs in the L + AA group were infused with a balanced amino acid mixture [34], prepared devoid of leucine, to maintain circulating amino acid concentrations at baseline fasting levels during the elevation in leucine. The infusion rate of the amino acid mixture (devoid of leucine) was progressively increased at 10 min intervals from 0 to 0.4, 0.6, 0.85, 1.5, 1.85, 2.25, 2.7 and 2.85 mL/kg•h, until the infusion rate of 2.85 mL/kg•h was reached, and maintained constant throughout as previously calculated in our laboratory [36]. We achieved the desired substrate and hormone concentrations targeted during our clamp procedure as described in our previous publication [35].

### Immunoblotting and immunoprecipitation (experiment 1 and 2)

Frozen longissimus dorsi muscle samples were homogenized and centrifuged at 10,000 *g* for 10 min at 4°C. The protein concentration was determined in the supernatant by the Bradford method [31]. Equal amounts (50 μg) of extracted protein were electrophoretically separated in polyacrylamide gels and transferred to polyvinylidene difluoride (PVDF) membrane (Bio-Rad, Hercules, CA), which were incubated with appropriate primary antibodies followed by appropriate secondary antibodies as previously described [33]. Blots were developed using an enhanced chemiluminescence kit (Amersham), visualized, and analyzed using a ChemiDoc-It Imaging System (UVP, Upland, CA). The protein abundance of each signaling components was normalized with β-actin abundance in the samples. Primary antibodies that were used in the immunoblotting were MuRF1, atrogin-1, β-actin (Santa Cruz Biotechnology, Santa Cruz, CA), rpS6, eIF4E, Lamp-2, ULK1, and LC3 (Cell Signaling Technology, Danvers, MA).

### Statistical analysis (experiment 1 and 2)

In Experiment 1, two-way ANOVA with Bonferroni post-test were used to determine the effects of age and each treatment and their interaction on the abundance and phosphorylation of protein degradation and synthesis signaling components. In Experiment 2, the statistical analysis was performed by one-way ANOVA with subsequent Tukey’s post-test. Each experiment used separate control animals. Probability values of *P* < 0.05 were considered statistically significant. Data are presented as mean ± SEM.

## Results

**Figure 1:**
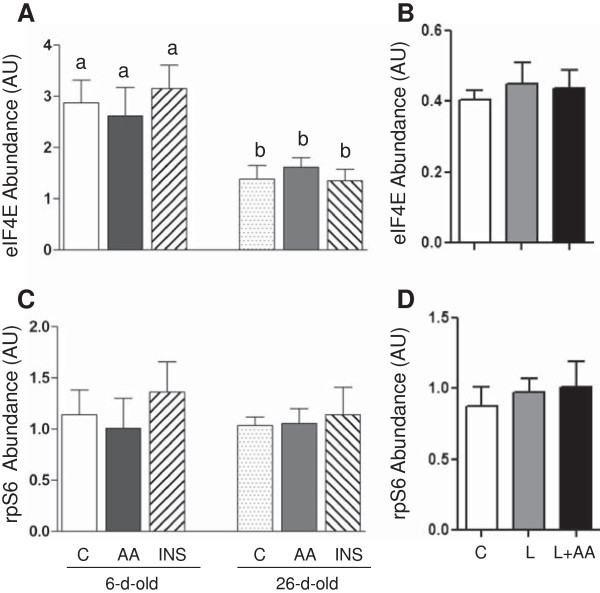
**The protein abundance of eIF4E and rpS6 in longissimus dorsi muscle in response to age and amino acids, insulin, and leucine infusion. (A)** Muscle eIF4E abundance from experiment 1 during euinsulinemic-euglycemic-euaminoacidemic (control; C), euinsulinemic-euglycemic-hyperaminoacidemic (AA), and hyperinsulinemic-euglycemic-euaminoacidemic (INS) clamps for 2 h in 6- and 26-d-old pigs. **(B)** Muscle eIF4E abundance from experiment 2 during euinsulinemic-euglycemic-euaminoacidemic-euleucinemic (C), euinsulinemic-euglycemic-hypoaminoacidemic-hyperleucinemic (L), and euinsulinemic-euglycemic-euaminoacidemic-hyperleucinemic (L+AA) clamps for 24 h in 5-d-old pigs. **(C)** Muscle rpS6 abundance from experiment 1 during C, AA, and INS clamps for 2 h in 6- and 26-d-old pigs. **(D)** Muscle rpS6 abundance from experiment 2 during C, L, and L+AA clamps for 24 h in 5-d-old pigs. Data are normalized with β-actin abundance and are expressed in arbitrary units (AU). Values are means ± SEM, n = 4–7. Values not sharing common symbols differ significantly (*P* < 0.05).

**Figure 2:**
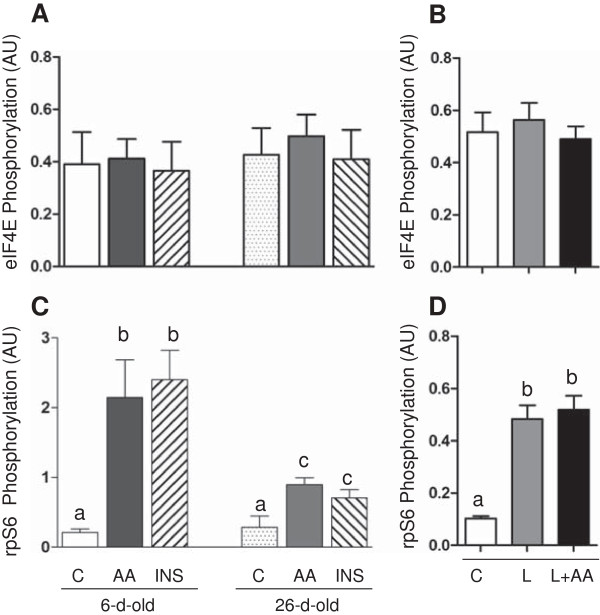
**The phosphorylation of eIF4E and rpS6 in longissimus dorsi muscle in response to age and amino acids, insulin, and leucine infusion. (A)** Muscle eIF4E phosphorylation from experiment 1 during euinsulinemic-euglycemic-euaminoacidemic (control; C), euinsulinemic-euglycemic-hyperaminoacidemic (AA), and hyperinsulinemic-euglycemic-euaminoacidemic (INS) clamps for 2 h in 6- and 26-d-old pigs. **(B)** Muscle eIF4E phosphorylation from experiment 2 during euinsulinemic-euglycemic-euaminoacidemic-euleucinemic (C), euinsulinemic-euglycemic-hypoaminoacidemic-hyperleucinemic (L), and euinsulinemic-euglycemic-euaminoacidemic-hyperleucinemic (L+AA) clamps for 24 h in 5-d-old pigs. **(C)** Muscle rpS6 phosphorylation from experiment 1 during C, AA, and INS clamps for 2 h in 6- and 26-d-old pigs. **(D)** Muscle rpS6 phosphorylation from experiment 2 during C, L, and L+AA clamps for 24 h in 5-d-old pigs. Data are normalized with eIF4E and rpS6 abundance and are expressed in arbitrary units (AU). Values are means ± SEM, n = 4–7. Values not sharing common symbols differ significantly (*P* < 0.05).

We previously reported that the abundance of many positive regulators of protein synthesis was significantly higher the younger the pig [33]. In this study, we extended our analysis to determine the effect of age on the abundance and the phosphorylation of two additional positive regulators of protein synthesis (rpS6 and eIF4E). As shown in Figure 1, the abundance of eIF4E, but not rpS6, was significantly higher in 6- compared to 26-d-old pigs (*P* < 0.05). As expected, short-term insulin or amino acid infusion had no effect on eIF4E or rpS6 abundance. Although neither insulin nor amino acids altered the phosphorylation of eIF4E, insulin and amino acids increased the phosphorylation of rpS6 and the response was greater in 6- than in 26-d-old pigs (*P* < 0.05) (Figure 2). Similar result was obtained on prolonged leucine infusion, where leucine, with or without amino acid replacement, had no effect on eIF4E phosphorylation but induced the phosphorylation of rpS6 (*P* < 0.05) (Figure 2).

**Figure 3:**
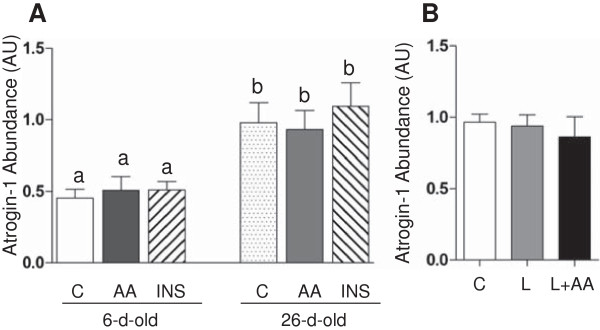
**The protein abundance of atrogin-1 in longissimus dorsi muscle in response to age and amino acids, insulin, and leucine infusion. (A)** Muscle atrogin-1 abundance from experiment 1 during euinsulinemic-euglycemic-euaminoacidemic (control; C), euinsulinemic-euglycemic-hyperaminoacidemic (AA), and hyperinsulinemic-euglycemic-euaminoacidemic (INS) clamps for 2 h in 6- and 26-d-old pigs. **(B)** Muscle atrogin-1 abundance from experiment 2 during euinsulinemic-euglycemic-euaminoacidemic-euleucinemic (C), euinsulinemic-euglycemic-hypoaminoacidemic-hyperleucinemic (L), and euinsulinemic-euglycemic-euaminoacidemic-hyperleucinemic (L+AA) clamps for 24 h in 5-d-old pigs. Data are normalized with β-actin abundance and are expressed in arbitrary units (AU). Values are means ± SEM, n = 4–7. Values not sharing common symbols differ significantly (*P* < 0.05).

**Figure 4:**
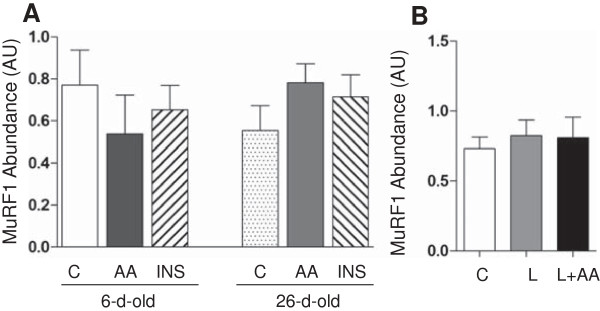
**The protein abundance of MuRF1 in longissimus dorsi muscle in response to age and amino acids, insulin, and leucine infusion. (A)** Muscle MuRF1 abundance from experiment 1 during euinsulinemic-euglycemic-euaminoacidemic (control; C), euinsulinemic-euglycemic-hyperaminoacidemic (AA), and hyperinsulinemic-euglycemic-euaminoacidemic (INS) clamps for 2 h in 6- and 26-d-old pigs. **(B)** Muscle MuRF1 abundance from experiment 2 during euinsulinemic-euglycemic-euaminoacidemic-euleucinemic (C), euinsulinemic-euglycemic-hypoaminoacidemic-hyperleucinemic (L), and euinsulinemic-euglycemic-euaminoacidemic-hyperleucinemic (L+AA) clamps for 24 h in 5-d-old pigs. Data are normalized with β-actin abundance and are expressed in arbitrary units (AU). Values are means ± SEM, n = 4–7. Values not sharing common symbols differ significantly (*P* < 0.05).

We determined the protein abundance of atrogin-1 and MuRF1 as indicators for the activation of the ubiquitin-proteasome pathway. As illustrated in Figure 3, the abundance of atrogin-1 was greater in 26- than in 6-d-old-pigs (*P* < 0.05). Neither short-term insulin nor amino acid infusion, or more prolonged leucine infusion, had an effect on the protein abundance of atrogin-1 (Figure 3). With regard to the abundance of the ubiquitin-proteasome component, MuRF1, there was no effect of age, acute amino acid or insulin infusion, or prolonged leucine administration (Figure 4).

**Figure 5:**
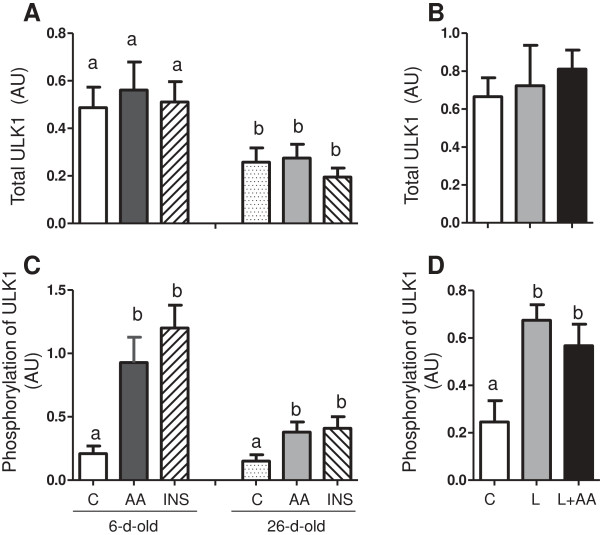
**The abundance and phosphorylation of ULK1 in longissimus dorsi muscle in response to age and amino acids, insulin, and leucine infusion. (A)** Muscle ULK1 abundance from experiment 1 during euinsulinemic-euglycemic-euaminoacidemic (control; C), euinsulinemic-euglycemic-hyperaminoacidemic (AA), and hyperinsulinemic-euglycemic-euaminoacidemic (INS) clamps for 2 h in 6- and 26-d-old pigs. **(B)** Muscle ULK1 abundance from experiment 2 during euinsulinemic-euglycemic-euaminoacidemic-euleucinemic (C), euinsulinemic-euglycemic-hypoaminoacidemic-hyperleucinemic (L), and euinsulinemic-euglycemic-euaminoacidemic-hyperleucinemic (L+AA) clamps for 24 h in 5-d-old pigs. **(C)** Muscle ULK1 phosphorylation from experiment 1 during C, AA, and INS clamps for 2 h in 6- and 26-d-old pigs. **(D)** Muscle ULK1 phosphorylation from experiment 2 during C, L, and L+AA clamps for 24 h in 5-d-old pigs. Data are expressed in arbitrary units (AU). Values are means ± SEM, n = 4–7. Values not sharing common symbols differ significantly (*P* < 0.05).

**Figure 6:**
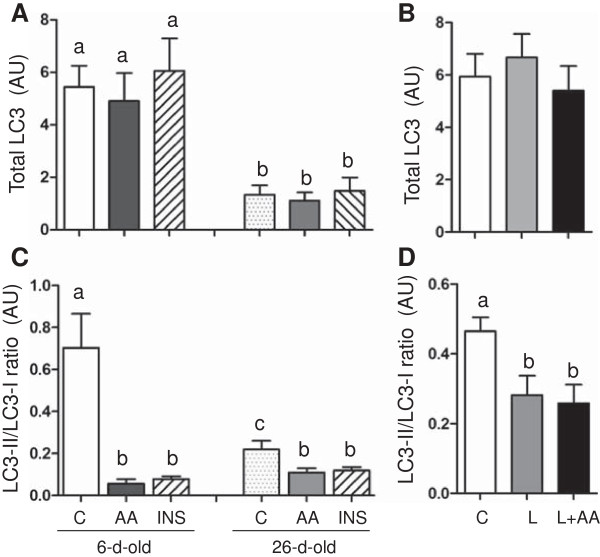
**The abundance of total LC3 and the ratio of LC3-II to LC3-I in longissimus dorsi muscle in response to age and amino acids, insulin, and leucine infusion. (A)** Muscle LC3 abundance from experiment 1 during euinsulinemic-euglycemic-euaminoacidemic (control; C), euinsulinemic-euglycemic-hyperaminoacidemic (AA), and hyperinsulinemic-euglycemic-euaminoacidemic (INS) clamps for 2 h in 6- and 26-d-old pigs. **(B)** Muscle LC3 abundance from experiment 2 during euinsulinemic-euglycemic-euaminoacidemic-euleucinemic (C), euinsulinemic-euglycemic-hypoaminoacidemic-hyperleucinemic (L), and euinsulinemic-euglycemic-euaminoacidemic-hyperleucinemic (L+AA) clamps for 24 h in 5-d-old pigs. **(C)** Muscle LC3-II to LC3-I ratio from experiment 1 during C, AA, and INS clamps for 2 h in 6- and 26-d-old pigs. **(D)** Muscle LC3-II to LC3-I ratio from experiment 2 during C, L, and L+AA clamps for 24 h in 5-d-old pigs. Data are expressed in arbitrary units (AU). Values are means ± SEM, n = 4–7. Values not sharing common symbols differ significantly (*P* < 0.05).

**Figure 7:**
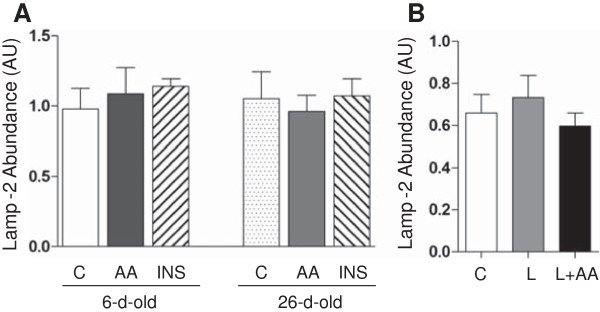
**The protein abundance of Lamp-2 in longissimus dorsi muscle in response to age and amino acids, insulin, and leucine infusion. (A)** Muscle Lamp-2 abundance from experiment 1 during euinsulinemic-euglycemic-euaminoacidemic (control; C), euinsulinemic-euglycemic-hyperaminoacidemic (AA), and hyperinsulinemic-euglycemic-euaminoacidemic (INS) clamps for 2 h in 6- and 26-d-old pigs. **(B)** Muscle Lamp-2 abundance from experiment 2 during euinsulinemic-euglycemic-euaminoacidemic-euleucinemic (C), euinsulinemic-euglycemic-hypoaminoacidemic-hyperleucinemic (L), and euinsulinemic-euglycemic-euaminoacidemic-hyperleucinemic (L+AA) clamps for 24 h in 5-d-old pigs. Data are normalized with β-actin abundance and are expressed in arbitrary units (AU). Values are means ± SEM, n = 4–7.

To study the effect of amino acids and insulin on the autophagy-lysosome system, we examined ULK1, the LC3-II/LC3-I ratio (a commonly used marker for autophagy) and the lamp-2 abundance (a marker for chaperon-mediated autophagy). First, we analyzed the total abundance and phosphorylation of ULK (Figure 5). ULK1 abundance was higher in 6- than in 26-d-old pigs (*P* < 0.05). Acute amino acid or insulin infusion or more prolonged leucine administration had no effect on ULK1 abundance. Amino acid- and insulin-induced phosphorylation of ULK1 was also higher in the younger pigs compared to their older counterparts (*P* < 0.05). Similarly, leucine infusion induced the phosphorylation of ULK1 (*P* < 0.05). We found also that the total abundance of LC3 decreased with age (*P* < 0.05) (Figure 6). Insulin and amino acids reduced the LC3-II/LC3-I ratio (*P* < 0.05), and this effect was greater in 6- than in 26-d-old pigs (*P* < 0.05). Likewise, leucine infusion, with or without amino acid replacement, decreased the LC3-II/LC3-I ratio (*P* < 0.05). With respect to the abundance of Lamp-2, none of the treatments had an effect on this component of the autophagy-lysosome system (Figure 7).

## Discussion

In order to support their rapid growth, neonates have higher protein turnover rates than adults. Since protein accretion takes place when the rate of postprandial protein synthesis is higher than the rate of protein degradation, the balance between these two processes is crucial. Although the molecular mechanisms by which protein synthesis and protein degradation are governed have been studied for a while, most of research has been performed in cell culture or has focused on mature individuals [11,12,29,30]. Here we studied the effects of insulin and amino acids, including leucine, on the abundance and activation of specific signaling components of the protein synthesis and degradation apparatus in neonatal pigs and how this changes with development.

Previously, we identified major signaling components that control protein synthesis in skeletal muscle of neonatal pigs, however, modulation by rpS6 and eIF4E was not examined [33]. Early studies show that rpS6 phosphorylation is involved in the regulation of global protein synthesis [37]. More recent studies using the transgenic mouse model indicate that rpS6 is crucial for the regulation of cell size [38]. Other observations suggest that following mitogenic or nutritional signals, rpS6 is involved in an efficient mechanism for the activation of protein synthesis that ensures balanced protein synthesis and controls energy wastage [39]. In the current study, we found that the abundance of rpS6 is not regulated by development. Likewise, neither amino acids nor insulin affected rpS6 abundance. Similar to its upstream regulator, S6K1 [8,14], the phosphorylation of rpS6 was stimulated by an acute infusion of insulin or amino acids that reproduced the rise in these agents with feeding. The more prolonged administration of leucine, in the presence of either hypoaminoacidemia or euaminoacidemia, also increased rpS6 phosphorylation, indicating that leucine was a primary driver for rpS6 stimulation. The anabolic effect of insulin and amino acids was higher in 6- than in 26-d-old pigs suggesting that the activation of rpS6 may play a significant role in the higher rate of protein synthesis during the neonatal period.

The translation initiation factor, eIF4E, is crucial for the regulation of cap-dependent translation that represents the standard mode of translation used by the vast majority of cellular mRNAs [40]. Over- and under-expression of eIF4E indicate that this initiation factor is important for the regulation of cell growth [41]. Furthermore, since eIF4E is overexpressed in several types of cancers, it is considered as a primary target for cancer drugs [42]. Interestingly, we found that eIF4E abundance is markedly elevated in 6- compared to 26-d-old pigs suggesting a role of eIF4E in the high muscle cell growth of young pigs. Studies also show that in several types of cells, the phosphorylation of eIF4E, induced by anabolic agents, is indispensable for protein synthesis and cell growth [43,44]. In the current study, neither the age nor anabolic agent treatments had any effect on the phosphorylation of eIF4E. This finding is consistent with those of Vary et al. [13] who found that the IGF-I-induced stimulation of protein synthesis occurs in the absence of changes in eIF4E phosphorylation. Nonetheless, the results show differential regulation of the translation initiation signaling proteins, rpS6 and eIF4E, with development. While the abundance of eIF4E decreases with age, its phosphorylation is unaffected by the anabolic agents tested. By contrast, the abundance of rpS6 does not change with age, but its phosphorylation in response to insulin and amino acids decreases with development.

The continual degradation and synthesis of protein, i.e., protein turnover, is crucial for homeostatic functions of normal cells [45]. Studies show that the ubiquitin-proteasome system plays a major role in the regulation of muscle protein degradation [29]. The abundance of the muscle-specific ubiquitin protein ligases (E3), atrogin-1/MAFbx and MuRF1, is crucial for skeletal muscle degradation in catabolic states [46]. The target protein substrates of atrogin-1 include MyoD, a transcriptional regulator which controls muscle size [46]. MuRF1 prefers structural protein such as titin and myosin light chain-1 (MLC1) as target proteins [46]. Taken together, these ligases regulate the substrate targets that play an important role in skeletal muscle growth. In this study, we determined the abundance of these ubiquitin ligases. We found that only atrogin-1 was affected by age. Although these results are consistent with the recent results of Orellana et al. [47], the differential response of the two ligases seems inconsistent with their functions as major players of protein degradation. The finding that these ligases are differentially expressed in certain experimental conditions is not uncommon. Frost et al. [48] found that the sepsis-induced increase in atrogin-1 mRNA expression, but not MuRF1, was completely blocked by IGF-I. Other studies show that the atrogin-1 mRNA expression, but not MuRF1, is increase by interleukin 6 (IL6) [49], and angiotensin II (ANG II) [50]. Conversely, the skeletal muscle MuRF1 mRNA expression, but not atrogin-1, is enhanced by exercise [46], inhibitor of nuclear factor kappa-B kinase subunit beta (IKKβ) gene deletion [51], and nuclear factor kB (NFkB) pathway activation [52].

Although atrogin-1 and MuRF1 were discovered more than a decade ago [53], their contributions to the activation of the ubiquitin-proteasome system are still controversial for several reasons [46]. First, their downstream substrates are not similar and in addition to regulating protein degradation, they also regulate other physiological functions [46]. Second, studies show that their functions are species-specific. For example, the mRNA expression of these ligases are increased in old rats but not in aged humans [54,55], and fasting increases atrogin-1 mRNA expression in mice but has no effect in humans [56,57]. Lastly, studies suggest that the activities of these ligases are altered without changes in their mRNA expression levels and that the primary role of these ligases is not to enhance muscle atrophy [50]. Moreover, another study showed that although muscle protein degradation is elevated in healthy older humans when compared to their healthy younger counterparts, the mRNA expression of these ligases is similar in both age groups [58]. Further studies are needed to evaluate the functions of these ubiquitin ligases and whether they are reliable markers for the ubiquitin-proteasome system in muscle [46]. Our observations also show that the abundance of these ligases was not affected by the acute rise in insulin or amino acids or more prolonged leucine administration. One possible explanation of the lack of effect on these ligases is the short length of the fasting time used in this study (12 h). This may not have been a sufficiently long enough fasting time to enhance the expression of these ligases and thus the animals may not have been sufficiently catabolic for amino acids or insulin to reverse this action. Several rodent studies show that prolonged fasting of 48 h or starvation significantly increased the mRNA expression of these two ligases [59,60]. This suggests that these ubiquitin ligases do not play critical roles in the regulation of protein balance during normal fasting-feeding cycles.

Autophagy plays a significant role in the catabolic process which is manifested by the degradation of protein aggregation and damaged organelles via the fusion between autophagosomes and lysosomes [18]. Although mTOR is crucial for the regulation of autophagy [61], the physiological role of autophagy in skeletal muscles has not been fully elucidated. Studies show that mTOR inhibits autophagy via inactivation of ULK1, a crucial component that resides upstream of LC3 [25]. We found that the ULK1 abundance was higher in younger pigs but did not change with short-term insulin or amino acid infusion or more prolonged administration of leucine. However, phosphorylation of ULK1 at Ser^757^ was increased in response to the acute rise in circulating insulin or amino acids, indicating a mTOR-induced inactivation of ULK1, and this effect was reduced with development. Likewise, more sustained leucine administration, in the presence of either hypoaminoacidemia or euaminoacidemia, also inactivated ULK1, likely by a mTOR-dependent mechanism.

During fasting and other stress conditions, autophagy has the vital role in maintaining the amino acid pool by digesting muscular protein and organelles [62]. In macroautophagy, LC3 is widely known as a marker of autophagosomes [63]. Under autophagy-inducing conditions, LC3-I (the cytosolic form) is processed and recruited to autophagosomes where the formation of lipid conjugated LC3-II attained by site specific proteolysis and lipidation near the C-terminus occurs [63]. Since the formation of LC3-II is positively correlated with autophagosome numbers, measuring the conversion of LC3-I to LC3-II by immunoblotting is considered to be a reliable assay to determine autophagic activity [63]. In the present study, short-term insulin or amino acid administration or more prolonged leucine supplementation suppressed the fasting-induced increase in skeletal muscle LC3-II protein levels. These anabolic effects were more robust in younger pigs. Interestingly, we observed decreased total LC3 protein levels due to development indicating that younger pigs have higher basal autophagy. This observation is consistent with our previous findings of higher rates of protein degradation in neonatal than in older rats [64].

We next sought to determine whether age, the acute infusion of amino acids or insulin, or the chronic administration of leucine affect chaperone-mediated autophagy by measuring the protein abundance of Lamp-2 [23,24]. We found that the abundance of Lamp-2 was not affected by any treatment. Although genetic studies [65] have shown that Lamp-2 is involved in regulating overall autophagy in skeletal muscle, our data suggest that during the neonatal period, chaperone-mediated autophagy does not play a major role in general autophagy.

## Conclusions

Throughout life, a delicate balance exists between protein synthesis and degradation that is essential for growth and normal health of humans and animals. Using neonatal pigs, we have sought to elucidate the molecular mechanisms by which protein synthesis and degradation are regulated, particularly in skeletal muscle. Hence, in these particular studies we focused on determining the effects of the acute rise in insulin and amino acids, as well as the more prolonged administration of leucine alone on the abundance and activation of signaling components which are important players of protein degradation and protein synthesis signaling pathways and their modulation by development. Our results, based on the ULK1 and LC3 data, and the data from Davis et al. [64] showing a development decline in the fractional rate of protein degradation support the notion that protein degradation activity is high during neonatal period to maintain the elevated protein turnover needed to sustain growth. Although the atrogin-1 and MuRF1 data do not support this hypothesis, the regulation of these ligases is still controversial and, thus more studies are needed. The results, along with previous findings [66], further suggest that eIF4E and rpS6 play crucial roles in ensuring high rates of protein synthesis in skeletal muscle of neonatal pigs. With respect to autophagy, the acute rise in insulin and amino acids, similar to that which occurs with feeding, as well as the more prolonged supplementation with leucine alone, irregardless of the circulating levels of other amino acids, had inhibitory effects on ULK1 and LC3-II. These responses are consistent with the suppressive effects of ULK1 and LC3-II on protein degradation. Likewise, all treatments had positive effect on the phosphorylation of rpS6, but not eIF4E, indicating that stimulation of eIF4E phosphorylation is not crucial for anabolic-induced activation of mRNA translation in skeletal muscle. Understanding how protein synthesis and protein degradation are regulated during the neonatal period is crucial for the development of new nutritional strategies that can support maximum growth of neonates.

## Abbreviations

4EBP-1: 4E-binding protein 1; ANG II: Angiotensin II; atrogin-1: A muscle-specific F-box protein highly expressed during muscle atrophy; BCAA: Branched-chain amino acids; CMA: Chaperone-mediated autophagy; eIF4E: Eukaryotic initiation factor 4E; IGF-I: Insulin-like growth factor-1; IKKβ: Inhibitor of nuclear factor kappa-B kinase subunit beta; IL6: Interleukin 6; Lamp-2: Lysosomal-associated membrane protein 2; LC3: Microtubule-associated protein light chain 3; NFkB: Nuclear factor kB; mTOR: Mammalian target of rapamycin; MuRF1: Muscle RING-finger protein-1; rpS6: Ribosomal protein S6; S6K1: Ribosomal protein S6 kinase 1; ULK1: Unc51-like kinase 1; UPS: Ubiquitin-proteasome system.

## Competing interests

The authors declare that they have no competing interests.

## Authors’ contributions

AS and TAD did the conception and design of the research; AS performed the experiments; AS analyzed the data; AS and TAD interpreted the results of the experiments; AS prepared the figures and drafted the manuscript; AS and TAD edited and revised the manuscript; and TAD had primary responsibility for the final content. Both authors approved the final version of the manuscript.
